# 4,5-Diaza-9*H*-fluoren-9-imine

**DOI:** 10.1107/S1600536810020441

**Published:** 2010-06-26

**Authors:** Hui Cang, Jin-Xiu Ji, Si-Qing Wang, Jin-Tang Wang

**Affiliations:** a Chemical and Biological Engineering College, Yancheng Institute of Technology, Yancheng 224051, People’s Republic of China; b Research & Development Center, Sinochem Jiangsu Corporation, Nanjing 210005, People’s Republic of China; c Department of Applied Chemistry, College of Science, Nanjing University of Technology, Nanjing 210009, People’s Republic of China

## Abstract

In the title compound, C_11_H_7_N_3_, the diaza­fluorene rings are almost coplanar with an r.m.s. deviation of 0.0160 Å. In the crystal structure, C—H⋯N hydrogen bonds link mol­ecules into sheets parallel to the *ab* plane. Mol­ecules are also stacked regularly along the *c* axis by a variety of π–π inter­actions with centroid–centroid distances in the range 3.527 (2)–3.908 (2) Å.

## Related literature

For the use of the title compound in synthesizing complexes with inter­esting photochemical properties and for the synthesis, see: Wang & Rillema (1997 ▶). For reference bond-length data, see: Allen *et al.* (1987 ▶).
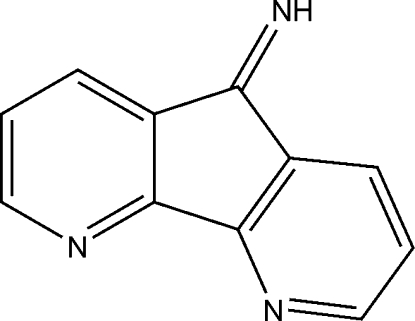


## Experimental

### 

#### Crystal data

C_11_H_7_N_3_
*M* *_r_* = 181.20
Monoclinic, 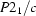

*a* = 10.008 (2) Å
*b* = 12.407 (3) Å
*c* = 6.8140 (14) Å
β = 99.74 (3)°
*V* = 833.9 (3) Å^3^
*Z* = 4
Mo *K*α radiation
μ = 0.09 mm^−1^
*T* = 293 K
0.30 × 0.10 × 0.10 mm

#### Data collection

Enraf–Nonius CAD-4 diffractometer
Absorption correction: ψ scan (North *et al.*, 1968 ▶) *T*_min_ = 0.973, *T*_max_ = 0.991
1638 measured reflections
1503 independent reflections
1010 reflections with *I* > 2σ(*I*)
*R*_int_ = 0.022
3 standard reflections every 200 reflections intensity decay: none

#### Refinement

*R*[*F* ^2^ > 2σ(*F* ^2^)] = 0.066
*wR*(*F* ^2^) = 0.194
*S* = 1.06
1503 reflections
127 parameters
40 restraints
H-atom parameters constrained
Δρ_max_ = 0.28 e Å^−3^
Δρ_min_ = −0.21 e Å^−3^

### 

Data collection: *CAD-4 Software* (Enraf–Nonius, 1985 ▶); cell refinement: *CAD-4 Software*; data reduction: *XCAD4* (Harms & Wocadlo, 1995 ▶); program(s) used to solve structure: *SHELXS97* (Sheldrick, 2008 ▶); program(s) used to refine structure: *SHELXL97* (Sheldrick, 2008 ▶); molecular graphics: *SHELXTL* (Sheldrick, 2008 ▶); software used to prepare material for publication: *SHELXTL*.

## Supplementary Material

Crystal structure: contains datablocks I, global. DOI: 10.1107/S1600536810020441/sj5007sup1.cif

Structure factors: contains datablocks I. DOI: 10.1107/S1600536810020441/sj5007Isup2.hkl

Additional supplementary materials:  crystallographic information; 3D view; checkCIF report

## Figures and Tables

**Table 1:** Hydrogen-bond geometry (Å, °)

*D*—H⋯*A*	*D*—H	H⋯*A*	*D*⋯*A*	*D*—H⋯*A*
C11—H11*A*⋯N3^i^	0.93	2.45	3.382 (4)	178
C4—H4*A*⋯N1^ii^	0.93	2.69	3.536 (4)	152

Symmetry codes: (i) 
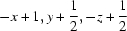
; (ii) 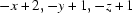.

**Fig. 1.**
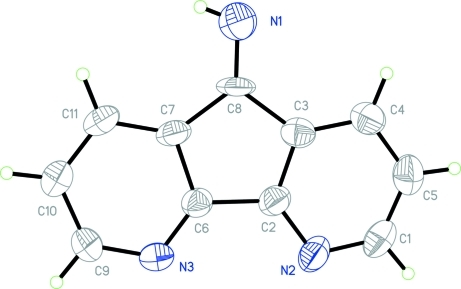
A drawing of the title molecular structure, with the atom-numbering scheme. Displacement ellipsoids are drawn at the 50% probability level.

**Fig. 2.**
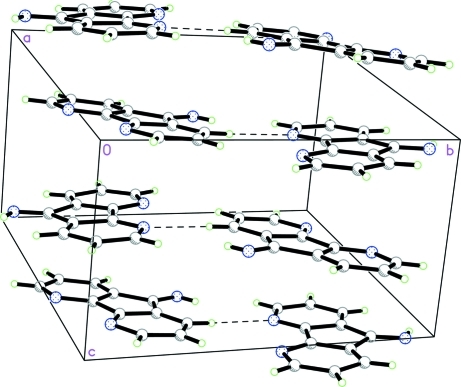
A packing diagram for (I). Hydrogen bonds are drawn as dashed lines.

## supplementary crystallographic information

### Comment

4,5-diazafluorene-9-imine is one of the important ligands, being utilized to synthesize complexes with interesting photochemical properties (Wang & Rillema, 1997). Here we report the crystal structure of the title compound, (I).

The molecular structure of (I) is shown in Fig. 1, and the selected geometric parameters are given in Table 1. The bond lengths and angles (Table 1) are within normal ranges (Allen *et al.*, 1987). The diazafluorene rings are almost coplanar with an rms deviation 0.0160 Å.

In the crystal structure C—H···N hydrogen bonds link molecules into sheets parallel to the *ab* plane, Table 1. An extensive system of π–π contacts stacks molecules in an obverse fashion down the *c* axis, Fig. 2, with Cg1···Cg1 = 3.876 (2) /%A, Cg2···Cg2 = 3.572 (2) /%A, Cg(3)···Cg3 = 3.908 (2) and Cg1···Cg2 3.776 (2) Å and 3.863 (2) Å. Symmetry operations x, 1/2-y, 1/2+z, and x, 1/2-y, -1/2+z; Cg1, Cg2 and Cg3 are the centroids of the C2,C3,C5,C7,C8; N2,C1,C2,C3,C4,C5 and N3,C6,C7,C10,C11 rings, respectively.

### Experimental

The title compound was synthesized by a method reported in literature (Wang & Rillema, 1997). Crystals were obtained by dissolving the compound  (2.0 g, 11.0 mmol) in ethyl acetate(50 ml), and evaporating the solvent slowly at room temperature for about 5 d.

### Refinement

H atoms were positioned geometrically, with N—H = 0.75 and C—H = 0.93Å for aromatic C–H, and constrained to ride on their parent atoms, with U_iso_(H) = xU_eq_(C/N), where x = 1.2 for aromatic H and x = 1.5 for the N–H.

### Crystal data

**Table:**

C_11_H_7_N_3_	*F*(000) = 376
*M**_r_* = 181.20	*D*_x_ = 1.443 Mg m^−^^3^
Monoclinic, *P*2_1_/*c*	Mo *K*α radiation, λ = 0.71073 Å
Hall symbol: -P 2ybc	Cell parameters from 25 reflections
*a* = 10.008 (2) Å	θ = 10–13°
*b* = 12.407 (3) Å	µ = 0.09 mm^−^^1^
*c* = 6.8140 (14) Å	*T* = 293 K
β = 99.74 (3)°	Block, colourless
*V* = 833.9 (3) Å^3^	0.30 × 0.10 × 0.10 mm
*Z* = 4	

### Data collection

**Table:**

Enraf–Nonius CAD-4 diffractometer	1010 reflections with *I* > 2σ(*I*)
Radiation source: fine-focus sealed tube	*R*_int_ = 0.022
graphite	θ_max_ = 25.3°, θ_min_ = 2.1°
ω/2θ scans	*h* = −11→11
Absorption correction: ψ scan (North *et al.*, 1968)	*k* = 0→14
*T*_min_ = 0.973, *T*_max_ = 0.991	*l* = 0→8
1638 measured reflections	3 standard reflections every 200 reflections
1503 independent reflections	intensity decay: none

### Refinement

**Table:**

Refinement on *F*^2^	Primary atom site location: structure-invariant direct methods
Least-squares matrix: full	Secondary atom site location: difference Fourier map
*R*[*F*^2^ > 2σ(*F*^2^)] = 0.066	Hydrogen site location: inferred from neighbouring sites
*wR*(*F*^2^) = 0.194	H-atom parameters constrained
*S* = 1.06	*w* = 1/[σ^2^(*F*_o_^2^) + (0.0948*P*)^2^ + 0.5792*P*] where *P* = (*F*_o_^2^ + 2*F*_c_^2^)/3
1503 reflections	(Δ/σ)_max_ < 0.001
127 parameters	Δρ_max_ = 0.28 e Å^−^^3^
40 restraints	Δρ_min_ = −0.21 e Å^−^^3^

### Special details

**Table:**

Geometry. All esds (except the esd in the dihedral angle between two l.s. planes) are estimated using the full covariance matrix. The cell esds are taken into account individually in the estimation of esds in distances, angles and torsion angles; correlations between esds in cell parameters are only used when they are defined by crystal symmetry. An approximate (isotropic) treatment of cell esds is used for estimating esds involving l.s. planes.
Refinement. Refinement of *F*^2^ against ALL reflections. The weighted *R*-factor wR and goodness of fit *S* are based on *F*^2^, conventional *R*-factors *R* are based on *F*, with *F* set to zero for negative *F*^2^. The threshold expression of *F*^2^ > σ(*F*^2^) is used only for calculating *R*-factors(gt) etc. and is not relevant to the choice of reflections for refinement. *R*-factors based on *F*^2^ are statistically about twice as large as those based on *F*, and *R*- factors based on ALL data will be even larger.

### Fractional atomic coordinates and isotropic or equivalent isotropic displacement parameters (Å^2^)

**Table:**

	*x*	*y*	*z*	*U*_iso_*/*U*_eq_	
N1	0.8062 (3)	0.5097 (2)	0.3404 (5)	0.0530 (8)	
H1	0.7491	0.5497	0.3246	0.079*	
N2	0.7922 (3)	0.1271 (2)	0.3022 (4)	0.0488 (8)	
N3	0.5012 (3)	0.2137 (2)	0.2363 (4)	0.0455 (7)	
C1	0.9253 (4)	0.1124 (3)	0.3376 (5)	0.0540 (10)	
H1A	0.9567	0.0418	0.3391	0.065*	
C2	0.7547 (3)	0.2304 (2)	0.3049 (5)	0.0410 (8)	
C3	0.8433 (3)	0.3171 (2)	0.3340 (4)	0.0389 (8)	
C4	0.9804 (4)	0.2998 (3)	0.3749 (5)	0.0508 (9)	
H4A	1.0425	0.3559	0.4027	0.061*	
C5	1.0202 (4)	0.1925 (3)	0.3720 (6)	0.0545 (10)	
H5A	1.1119	0.1751	0.3935	0.065*	
C6	0.6122 (3)	0.2709 (2)	0.2711 (4)	0.0360 (7)	
C7	0.6173 (3)	0.3850 (2)	0.2811 (5)	0.0422 (8)	
C8	0.7604 (3)	0.4188 (2)	0.3239 (5)	0.0441 (8)	
C9	0.3856 (3)	0.2703 (3)	0.2096 (5)	0.0496 (9)	
H9A	0.3044	0.2324	0.1837	0.060*	
C10	0.3791 (3)	0.3820 (3)	0.2181 (6)	0.0546 (10)	
H10A	0.2955	0.4167	0.2002	0.066*	
C11	0.4973 (3)	0.4412 (3)	0.2531 (5)	0.0505 (9)	
H11A	0.4959	0.5161	0.2576	0.061*	

### Atomic displacement parameters (Å^2^)

**Table:**

	*U*^11^	*U*^22^	*U*^33^	*U*^12^	*U*^13^	*U*^23^
N1	0.0453 (17)	0.0273 (15)	0.083 (2)	−0.0112 (13)	0.0014 (15)	0.0041 (14)
N2	0.0498 (18)	0.0401 (16)	0.0546 (17)	0.0120 (14)	0.0033 (13)	0.0013 (13)
N3	0.0466 (17)	0.0278 (14)	0.0614 (17)	−0.0041 (12)	0.0071 (13)	−0.0015 (12)
C1	0.058 (2)	0.041 (2)	0.063 (2)	0.0158 (18)	0.0088 (17)	0.0032 (17)
C2	0.0445 (18)	0.0317 (17)	0.0454 (18)	0.0036 (14)	0.0032 (14)	−0.0001 (14)
C3	0.0430 (18)	0.0370 (17)	0.0380 (15)	−0.0060 (14)	0.0105 (13)	0.0004 (13)
C4	0.045 (2)	0.046 (2)	0.059 (2)	−0.0079 (16)	0.0011 (16)	0.0065 (16)
C5	0.0399 (19)	0.065 (3)	0.058 (2)	0.0084 (18)	0.0070 (15)	0.0018 (18)
C6	0.0387 (16)	0.0285 (16)	0.0390 (16)	−0.0005 (13)	0.0011 (12)	−0.0013 (12)
C7	0.0478 (19)	0.0246 (16)	0.0516 (18)	−0.0031 (14)	0.0013 (14)	0.0001 (13)
C8	0.0497 (19)	0.0282 (16)	0.0536 (19)	−0.0097 (15)	0.0062 (15)	−0.0024 (14)
C9	0.0339 (18)	0.045 (2)	0.068 (2)	−0.0037 (15)	0.0030 (15)	−0.0008 (17)
C10	0.045 (2)	0.040 (2)	0.079 (3)	0.0097 (17)	0.0094 (17)	−0.0004 (18)
C11	0.056 (2)	0.0250 (17)	0.069 (2)	0.0054 (16)	0.0049 (17)	0.0010 (16)

### Geometric parameters (Å, °)

**Table:**

N1—C8	1.216 (4)	C4—C5	1.391 (5)
N1—H1	0.7500	C4—H4A	0.9300
N2—C1	1.325 (4)	C5—H5A	0.9300
N2—C2	1.336 (4)	C6—C7	1.417 (4)
N3—C6	1.305 (4)	C7—C11	1.373 (4)
N3—C9	1.340 (4)	C7—C8	1.473 (4)
C1—C5	1.367 (5)	C9—C10	1.389 (5)
C1—H1A	0.9300	C9—H9A	0.9300
C2—C3	1.386 (4)	C10—C11	1.379 (5)
C2—C6	1.493 (4)	C10—H10A	0.9300
C3—C4	1.371 (5)	C11—H11A	0.9300
C3—C8	1.505 (4)		
			
C8—N1—H1	109.5	N3—C6—C7	125.1 (3)
C1—N2—C2	113.9 (3)	N3—C6—C2	127.3 (3)
C6—N3—C9	115.4 (3)	C7—C6—C2	107.6 (3)
N2—C1—C5	125.4 (3)	C11—C7—C6	118.5 (3)
N2—C1—H1A	117.3	C11—C7—C8	132.9 (3)
C5—C1—H1A	117.3	C6—C7—C8	108.6 (3)
N2—C2—C3	124.9 (3)	N1—C8—C7	128.4 (3)
N2—C2—C6	125.7 (3)	N1—C8—C3	125.3 (3)
C3—C2—C6	109.4 (3)	C7—C8—C3	106.3 (2)
C4—C3—C2	120.1 (3)	N3—C9—C10	124.3 (3)
C4—C3—C8	131.7 (3)	N3—C9—H9A	117.9
C2—C3—C8	108.0 (3)	C10—C9—H9A	117.9
C3—C4—C5	115.2 (3)	C11—C10—C9	119.5 (3)
C3—C4—H4A	122.4	C11—C10—H10A	120.2
C5—C4—H4A	122.4	C9—C10—H10A	120.2
C1—C5—C4	120.4 (3)	C7—C11—C10	117.3 (3)
C1—C5—H5A	119.8	C7—C11—H11A	121.3
C4—C5—H5A	119.8	C10—C11—H11A	121.3
			
C2—N2—C1—C5	−0.8 (5)	N3—C6—C7—C11	0.2 (5)
C1—N2—C2—C3	2.3 (5)	C2—C6—C7—C11	179.6 (3)
C1—N2—C2—C6	−179.7 (3)	N3—C6—C7—C8	179.7 (3)
N2—C2—C3—C4	−4.0 (5)	C2—C6—C7—C8	−0.9 (4)
C6—C2—C3—C4	177.8 (3)	C11—C7—C8—N1	−1.9 (6)
N2—C2—C3—C8	179.3 (3)	C6—C7—C8—N1	178.7 (4)
C6—C2—C3—C8	1.1 (3)	C11—C7—C8—C3	−179.1 (3)
C2—C3—C4—C5	3.7 (5)	C6—C7—C8—C3	1.5 (4)
C8—C3—C4—C5	179.5 (3)	C4—C3—C8—N1	5.0 (6)
N2—C1—C5—C4	1.0 (6)	C2—C3—C8—N1	−178.9 (3)
C3—C4—C5—C1	−2.4 (5)	C4—C3—C8—C7	−177.8 (3)
C9—N3—C6—C7	−0.2 (5)	C2—C3—C8—C7	−1.6 (3)
C9—N3—C6—C2	−179.5 (3)	C6—N3—C9—C10	−0.4 (5)
N2—C2—C6—N3	1.0 (5)	N3—C9—C10—C11	1.0 (6)
C3—C2—C6—N3	179.3 (3)	C6—C7—C11—C10	0.4 (5)
N2—C2—C6—C7	−178.4 (3)	C8—C7—C11—C10	−179.0 (3)
C3—C2—C6—C7	−0.1 (3)	C9—C10—C11—C7	−0.9 (5)

### Hydrogen-bond geometry (Å, °)

**Table:**

*D*—H···*A*	*D*—H	H···*A*	*D*···*A*	*D*—H···*A*
C11—H11A···N3^i^	0.93	2.45	3.382 (4)	178
C4—H4A···N1^ii^	0.93	2.69	3.536 (4)	152

Symmetry codes: (i) −*x*+1, *y*+1/2, −*z*+1/2; (ii) −*x*+2, −*y*+1, −*z*+1.
